# A joinpoint and age–period–cohort analysis of ocular cancer secular trends in Iran from 2004 to 2016

**DOI:** 10.1038/s41598-022-26349-x

**Published:** 2023-01-19

**Authors:** Mohammad Abolhosseini, Zahra Khorrami, Sare Safi, Mohammad Esmaeil Akbari, Seyed Mohamadmehdi Moshtaghion, Seyed Farzad Mohammadi, Mozhgan Rezaei Kanavi, Saeed Karimi

**Affiliations:** 1 Ophthalmic Epidemiology Research Center, Research Institute for Ophthalmology and Vision Science, Shahid Beheshti University of Medical Sciences, No. 23, Paidarfard St., Pasdaran Ave., Tehran, Iran; 2 Ocular Tissue Engineering Research Center, Research Institute for Ophthalmology and Vision Science, Shahid Beheshti University of Medical Sciences, No 23, Paydar fard st, Pasdaran ave, Tehran, Iran; 3 Cancer Research Center, Shahid Beheshti University of Medical Sciences, Tehran, Iran; 4 Ophthalmic Research Center, Research Institute for Ophthalmology and Vision Science, Shahid Beheshti University of Medical Sciences, No 23, Paydar fard st, Pasdaran ave, Tehran, Iran; 5 Translational Ophthalmology Research Center, Farabi Eye Hospital, Tehran University of Medical Sciences, Tehran, Iran

**Keywords:** Epidemiology, Eye cancer

## Abstract

Investigating secular trends of ocular cancer registration in Iran. After acquiring Iranian national population-based cancer registry data, trends of age-standardised incidence rates (ASIR) of ocular cancers and annual percent changes (APC) between 2004 and 2016 were analysed in age groups, gender, topography and morphology types with joinpoint regression analysis. Age, period, and cohort effects on incidence rates were estimated by age–period–cohort model. Geographic distribution of ASIR was assessed using GIS. Overall ASIR of ocular cancers was 16.04/100,000 (95% CI 15.77–16.32). Joinpoint regression analysis showed a significant increase of ASIR between 2004 and 2009 for males (APC = 5.5, 95% CI  0.9–10.2), ages over 50 years (APC = 5.2, 1.2–9.4), skin/canthus/adnexal cancers (APC = 4.2, 0.8–7.7), and carcinomas/adenocarcinomas (APC = 4.3, 0.6–8.1); however, between 2009 and 2016 a declining trend was observed in all investigated variables. ASIR of retinoblastoma was significantly increased (averaged APC = 20.7, 9–33.7) between 2004 and 2016. age–period–cohort analyses showed that incidence rates of ocular cancers significantly increased with aging, time periods, and birth cohort effects (p < 0.001). ASIR varied from 6.7/100,000 to 21.7/100,000 in Iran. Excepting retinoblastoma, all ocular cancer incidence trends were downward over a 13-year period; however, it was increasing between 2004 and 2009 cancer. ASIR was significant aging in Iran.

## Introduction

Ocular cancers are rare and account for approximately 0.2% of all diagnosed cancers in Western countries^1,2^. However, it is the only ocular disease that threatens life in addition to sight which is difficult to diagnose at early stages because of its rarity. Moreover, the global burden of ocular cancer imposes greater economic costs on the society in addition to the patients’ lives^3,4^. The annual incidence rates of ocular cancer in females in the U.S. and Asian countries were approximately 0.6 and 0.2–0.5 per 100,000 population, respectively. In males, it was 0.5 and 0.1–0.5 per 100,000 population, in that order^5^.

The etiology of ocular cancers is still uncertain. It is believed that there is a correlation between demographic and environmental conditions and the incidence of ocular cancer^6^. In several studies, the incidence of most ocular cancers was increased with advancing age^7–9^. Environmental factors such as ultraviolet (UV) B irradiation have been associated with the increased risk of uveal melanoma and ocular surface squamous neoplasia (OSSN)^7,10^. Race/ethnicity, genetic and molecular changes, and patients’ susceptibility factors have also been associated with most ocular cancers^11,12^.

Late diagnosis of ocular cancer is a major issue in low middle-income countries^13^, since early diagnosis and appropriate treatment are critical for optimal visual outcomes and survival rates^14^. In developing countries, due to the lack of appropriate facilities, most ocular cancers are diagnosed at the advanced stage, and the main goal of treatment in such cases would be preventing patients’ mortality ^15,16^. However, in developed countries, where there is adequate access to health care and early diagnosis available to patients, the goal of treatment has changed from reducing mortality to preserving vision^17–19^.

A quantitative assessment of cancer epidemiological measures would be necessary for the development of effective cancer control plans^20^. Population-based cancer registries provide the most practical data on cancer incidence and can enhance patient care by engaging clinicians along the research process^21^. The incidence of ocular cancer in both developed^22^ and developing countries^23–27^had an increasing trend; however, it was more significant in developing countries. There is a lack of comprehensive analysis of recent incidence trends of ocular cancers in Iran. The Iranian National Population-based Cancer Registry (INPCR) regularly collects and analyses cancer patient data at the national level for all 31 provinces of Iran since 1998, which is crucial for analysing the situation and planning cancer control policies in Iran^28^. According to the latest report of the INPCR (2016), the incidence rate and ASIR of ocular cancer were 0.36 and 0.41, respectively (0.23% of total body cancers)^29^. In this study, we investigated the incidence secular trend of all types of ocular cancers in Iran from 2004 to 2016, after acquiring the INPCR data, stratified by gender, age group, topography and morphology types of cancers using the joinpoint and age-period-cohort models. We also described the age, gender, and geographic-based variations in the registration of ocular cancers in Iran.

## Results

### Demographic and cancer characteristics

From 2004 to 2016, a total of 12,824 ocular cancers were diagnosed and recorded with the three specific ICD-O-3 codes, by INPCR. The overall incidence proportion of patients with all types of ocular cancers during the 2004-to-2016 period was 16.04 per 100,000 population (95% Confidence Interval (CI), 15.77–16.32). The overall incidence proportion of males was approximately 1.32 times higher than that of females (18.22 [95% CI, 17.81–18.64] versus (13.81 [95% CI, 13.44–14.18] per 100,000 population). The relative risk of all ocular cancer cases in the age group > 50 years to the age group ≤ 50 years was 13.1. The incidence proportion and relative risk according to patient characteristics were shown in Table 1.

**Table 1:** Frequency of ocular cancers in Iran during the 2004–2016 period.

Variable	Total (n = 12,824)				Men (n = 7380)				Women (n = 5444)				P-value
	N	%	IP	RR	N	%	IP	RR	N	%	IP	RR	
Age at diagnosis (mean ± SD)	57.6 ± 22.3				58.6 ± 22.2				56.3 ± 22.3				*0.075
**Age group**													
≤ 50 year	3354	26.4	4.90	Ref	1812	24.7	5.5	Ref	1542	28.6	4.5	Ref	**P < 0.001
> 50 year	9370	73.6	64.4	13.1	5516	75.3	76.2	13.8	3854	71.4	52.7	11.7	
**Topography**													
Skin / canthus / adnexa	9723	75.8	11.9	91.5	5505	74.6	6.7	111.6	4218	77.5	5.16	73.7	**P < 0.001
Intraocular	775	6	0.95	7.3	418	5.7	0.51	8.5	357	6.6	0.44	6.3	
Ocular surface	1125	8.8	1.38	10.6	805	10.9	0.98	16.3	320	5.9	0.39	5.6	
Orbit	565	4.4	0.69	5.3	308	4.2	0.38	6.3	257	4.7	0.31	4.4	
Unspecified	532	4.1	0.65	5	297	4	0.36	6	235	4.3	0.29	4.1	
Lacrimal gland and duct	104	0.8	0.13	Ref	47	0.6	0.06	Ref	57	1	0.07	Ref	
**Morphology**													
Carcinomas / adenocarcinomas	10,755	83.9	5.47	136.75	6283	85.1	7.68	256	4472	82.1	5.47	136.75	**P < 0.001
Melanomas	611	4.8	0.35	8.75	323	4.4	0.39	13	288	5.3	0.35	8.75	
Retinoblastoma	602	4.7	0.34	8.5	320	4.3	0.39	13	282	5.2	0.34	8.5	
Lymphoma	266	2.1	0.15	3.75	141	1.9	0.17	5.67	125	2.3	0.15	3.75	
Sarcoma	236	1.8	0.13	3.25	131	1.8	0.16	5.33	105	1.9	0.13	3.25	
Neurologic	59	0.5	0.04	Ref	27	0.4	0.03	Ref	32	0.6	0.04	Ref	
Others (mixed, rare and unspecified)	295	2.3	0.36	9	155	2.1	0.19	6.33	140	2.6	0.17	4.25	

*IP* incidence proportion; the incidence rate is the number of ocular cancer cases (per 100,000 population), *RR* relative risk; the relative risk is the ratio of the two-incidence proportion, *SD* Standard deviation.

The P-value for differences between percentages of groups.

*The t- test was used to test the difference of the mean age at diagnosis between genders.

**The Chi square test is used to test hypotheses about distribution of age groups, topography and morphology types between genders.

The "age at diagnosis" variable had 0.7% missing.

### Incidence trends of ocular cancer by age at diagnosis, gender, topography, and morphology types

**Figure 1:**
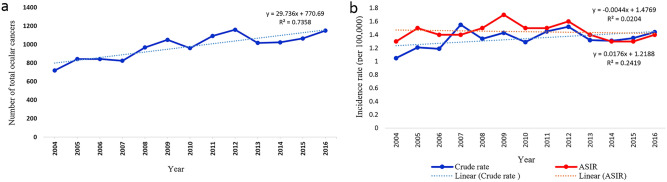
Number, crude incidence rates and age-standardised (world population, per 100,000 population) incidence rates for ocular cancers in Iran from 2004 to 2016.

**Figure 2:**
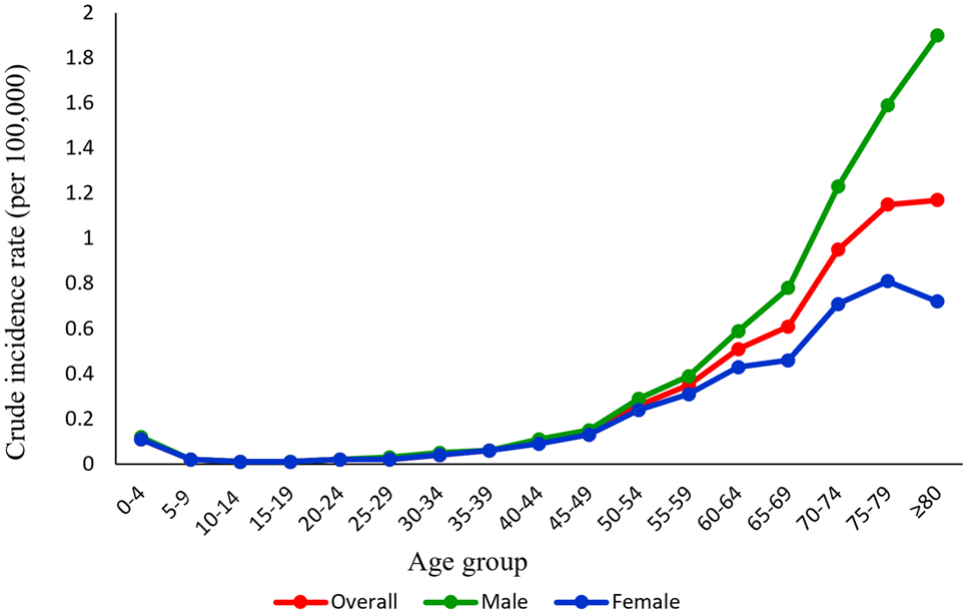
Crude incidence rates for ocular cancers by age groups in Iran from 2004 to 2016.

Figure 1 shows trends of number, crude incidence rates and age-standardised incidence rates for ocular cancers from 2004 to 2016. These trends were increasing significantly for the incidence number of ocular cancers (P < 0.001), and were borderline -for the crude incidence rates (P = 0.088). Figure 2 shows the incidence rate of all ocular cancers by age group from 2004 to 2016. At ages older than 80 years, the incidence rate of ocular cancers in Iran was highest among the male patients (1.9 per 100,000). However, females in the age group 75 to 79 years had the highest incidence of ocular cancers from 2004 to 2016 (0.81 per 100, 000).

**Figure 3:**
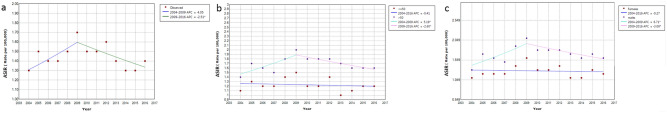
The trend in age-standardised (world population, per 100,000 population) incidence rates for (**a**) ocular cancers overall, (**b**) by age group, and (**c**) by gender in Iran between 2004 and 2016 (trend modeled with joinpoint regression).

The annual crude incidence rates of ocular cancers in age groups, both genders, topography and morphology types in Iran between 2004 and 2016 were shown in Supplementary Fig. S1 and Table S1. The ASIR of ocular cancers in all patients had its lowest value in 2004 (ASIR: 0.66 per 100,000 population) and peaked in 2009 (ASIR: 0.91 per 100,000 population). The trend in ASIR from 2004 to 2009 was a non-significant increasing trend (APC = 4.1, 95% CI, −1.3 to 93.7, p = 0.121). However, there was a significant decreasing trend of ASIR of ocular cancers from 2009 to 2016 (APC = −2.5, 95%CI, −4.6 to −0.4, p = 0.028) (Table 2; Fig. 3a). The trend of ASIR of ocular cancers in the age group over 50 years from 2004 to 2009 was a significantly increasing trend (APC = 5.2, 95% CI 1.2–9.4, p = 0.017). However, the ASIR of ocular cancers in the age group over 50 years decreased significantly from 2009 to 2016 (APC = −2.6, 95% CI, −4.1 to −1.1, p = 0.004) (Table 2; Fig. 3b). The trend of ASIR of ocular cancers in male patients from 2004 to 2009 was a significantly increasing trend (APC = 6.7, 95% CI 1—12.7, p = 0.022). In addition, the ASIR of ocular cancers in male gender decreased significantly from 2009 to 2016 (APC = −3.1, 95% CI −5.5 to −0.6, p = 0.015) (Table 2; Fig. 3c).

**Table 2:** Annual age-adjusted incidence rates of ocular cancers in Iran between 2004 and 2016.

	Trend 1		Trend 2		2004–2016
	Period	APC ( 95% CI)	Period	APC (95% CI)	AAPC (95% CI)
**Overall**	2004–2009	4.1 (−1.3, 93.7)	2009–2016	−2.5 (−4.6, −0.4)*	0.2 (−2, 2.4)
**Age group**					
≤ 50 year**	2004–2016	−0.4 (−1.9, 1.1)			−0.4 (−1.9, 1.1)
> 50 year	2004–2009	5.2 (1.2, 9.4)*	2009–2016	−2.6 (−4.1, −1.1)*	−0.6 (−1, 2.2)
**Gender**					
Males	2004–2009	6.7 (1, 12.7)*	2009–2016	−3.1 (−5.5, −0.6)*	0.9 (−1.4, 3.2)
Females**	2004–2016	−0.3 (−2.3, 1.8)			−0.3 (−2.3, 1.8)
**Topography**					
Orbit	2004–2012	10.8 (1.4, 21)*	2012–2016	−5.9 (−17.7, 7.6)	4.9(−1.5, 11.7)
Lacrimal Gland and Duct**	2004–2016	−0.5 (−4.7, 4)			−0.5 (−4.7, 4)
Intraocular**	2004–2016	−4 (−6.4, −1.5)*			−4 (−6.4, −1.5)*
Ocular Surface	2004–2009	4 (−4.5, 13.2)	2009–2016	−10.4 (−16.8, −3.6)*	−4.7(−9.1, −0.1)*
Skin / Canthus / Adnexa	2004–2009	4.2 (0.8, 7.7)*	2009–2016	−2.4 (−4.1, −0.8)*	0.3 (−1.2, 1.7)
Unspecified	2004–2008	23.2 (3.2, 47.1)*	2008–2016	−3.4 (−6.7, −0.001)*	4.8 (−0.7, 10.6)
**Morphology**					
Carcinoma / Adenocarcinoma	2004–2009	4.3 (0.6, 8.1)*	2009–2016	−4.2 (−6.1, −2.2)*	−0.7 (−2.3, 0.9)
Lymphoma	2004–2009	15.1 (−3.5, 37.4)	2009–2016	−26.6 (−44.3, −3.2)*	−11.4 (−23.8, 3)
Melanoma	2004–2010	6.2 (−3.2, 16.5)	2010–2016	−10 (−18.1, −1.1)*	−2.2 (−7.6, 3.4)
Sarcoma	2004–2007	3.1 (−13.5, 22.8)	2007–2016	−16.1 (−22.1, −9.5)*	−11.7 (−16.8, −6.1)*
Neurologic	2004–2007	41.4 (16.5, 71.6)*	2007–2016	−36.9 (−45.4, −27.2)*	−22.8 (−30.2, −14.6)*
Retinoblastoma	2004–2013	25.9 (8.5, 46.1)*	2013–2016	6.4 (−10.5, 26.5)	20.7 (9, 33.7)*
Others (Mixed, Rare and Unspecified)	2004–2011	−0.6(−6.2, 5.2)	2011–2016	−44.8 (−66.1, −10.3)*	−22.3 (−34.7, −7.4)*

*APC* annual percentage change, *AAPC* average annual percent change, *CI* confidence interval.

*The annual percent changes (APC) were significantly different from 0 for a specific trend (P-value < 0.05).

**The linear model without a joinpoint best describes the trend.

**Figure 4:**
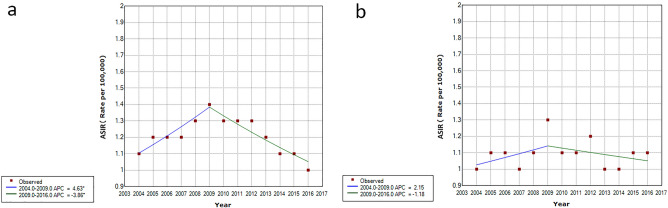
The trend in age-standardised (world population, per 100,000 population) incidence rates of carcinoma and adenocarcinoma (**a**) and skin canthus adnexa (**b**) in Iran between 2004 and 2016 (trend modeled with joinpoint regression).

Figure 4 shows the trend of ASIR per 100,000 for the most common topography (skin/canthus/adnexa) and morphology (carcinomas/adenocarcinomas) of ocular cancers. The trends of ASIR of skin/canthus/adnexal cancers, and of carcinomas/adenocarcinomas from 2004 to 2009 were significantly increasing (APC = 4.2 and APC = 4.3, respectively). In addition, there was a significant decreasing trend in ASIR from 2009 to 2016 (APC = −2.4 and APC = −4.2, respectively) for skin/canthus/adnexal cancers, and for carcinomas/adenocarcinomas. The AAPC of age-adjusted incidence rates of ocular cancers by topography and morphology types from 2004 to 2016 were also reported in Table 2. The joinpoint models showed a significant decreasing trend in intraocular (AAPC = −4, 95% CI −6.4 to −1.5), ocular surface (AAPC = −4.7, 95% CI −9.1 to −0.1) and sarcomatous (AAPC = −11.7, 95% CI −16.8 to −6.1) cancers. However, there was a significant and surprising increase rate of retinoblastoma (APPC = 20.7, 95% CI 9–33.7) from 2004 to 2016 in Iran.

### Age-period-cohort analysis

**Figure 5:**
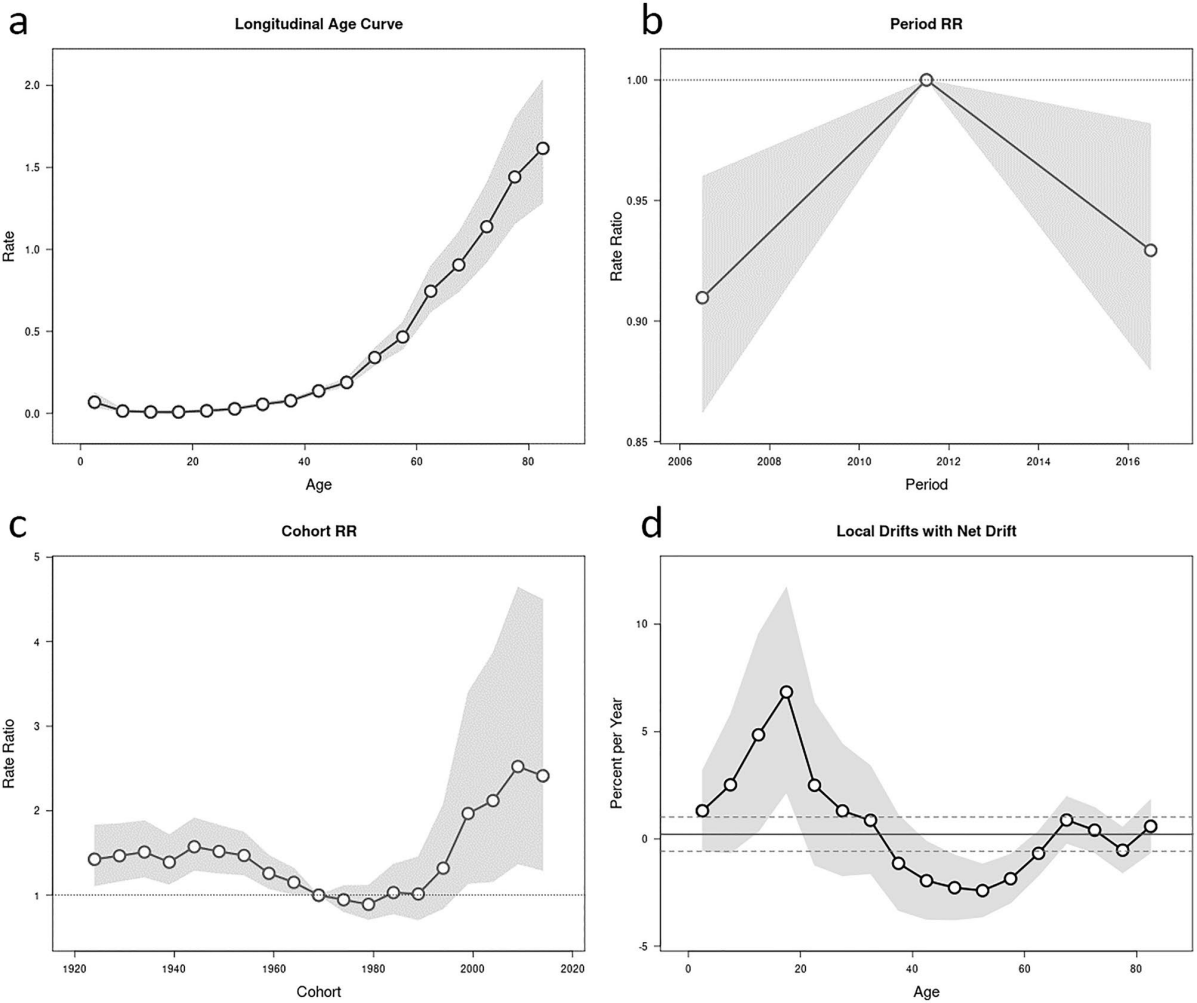
Age-period-cohort parameters and functions for the incidence of ocular cancer incidence, including (**a**) longitudinal age curve, (**b**) period RR, (**c**) cohort RR, and (**d**) local drifts with net drift [the shaded gray regions represent the 95% confidence interval. The solid and dotted horizontal lines in (**d**) represent net drift and the 95% confidence interval; *RR* rate ratio].

Age-period-cohort modeling showed the influence of age, period, and cohort on incidence trends (p < 0.001). The longitudinal age curve is displayed in Fig. 5a. The risk of developing ocular cancer increased with age and was highest in patients older than 80 years (1.17 per 100,000, 95%CI, 1.12–1.24). The effects of the period and cohort on ocular cancers were displayed in Fig. 5b,c. The rate ratio (RR) was higher in the third period than in the first period (RR = 0.93 in 2016 versus RR = 0.91 in 2006). The RR was highest in the 2009 birth cohort (RR = 2.52, 95%CI 1.37 to 4.64) (Fig. 5c). Figure 5d demonstrates the local drift values, which were quite modest, with an overall net drift of 0.21% per year (95%CI, -0.58 to 1.01). Local drift values were below zero at 37.5 to 62.5 years and 77.5 years whereas these values were above zero at other ages, with a peak of 6.84% per year (95%CI 2.17–11.72%) in patients older than 17.5 years.

### Geographic distribution of ASIR of ocular cancers

**Figure 6:**
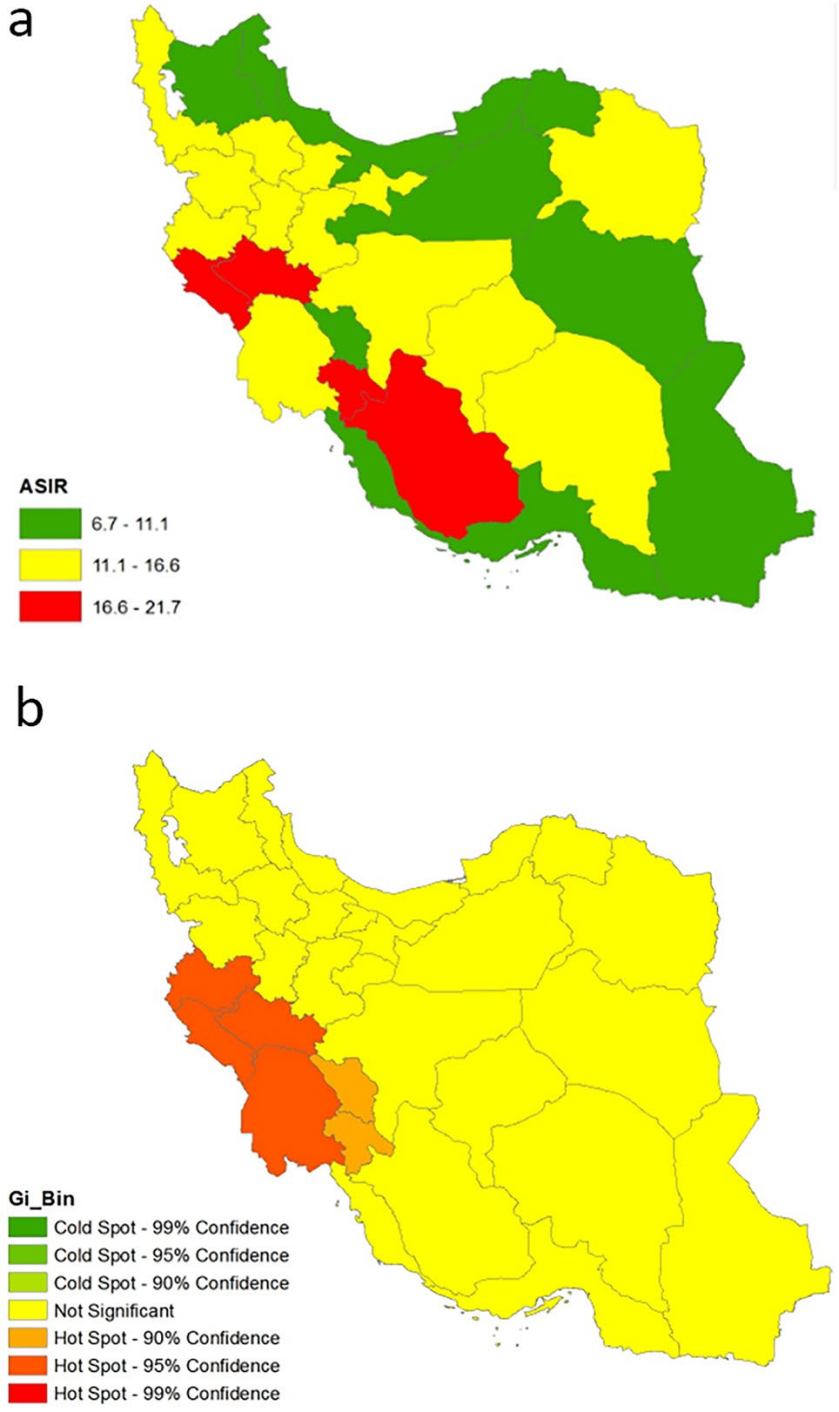
The Iranian province's age-standardised incidence rates hot spots and cold spots for all ocular cancers from 2004 to 2016. Reference: “This map was created using ArcMap (V. 10.3) software by Esri. ArcGIS® and ArcMap™ are the intellectual property of Esri and are used herein under license. Copyright © Esri. All rights reserved. For more information about Esri® software, please visit https://www.esri.com”.

The geographic distribution at the provincial level showed that the ASIR of ocular cancers ranged from 6.7 per 100,000 in Hormozgan, a province in Southern Iran, to 21.7 per 100,000 in Ilam, a province in Western Iran. The Moran coefficient was 0.34 (P < 0.001), indicating a spatial autocorrelation. The provinces with higher ASIR were located in the West of the country. We observed a hot spot for geographic distribution of ASIR of ocular cancers in the West of Iran (Fig. 6).

## Discussion

In this study, we showed the registration trends of various ocular cancers in Iran based on data from the INPCR, which includes the entire population of Iran. According to the literature review and to the best of our knowledge, the current study is the first to investigate the trend of whole ocular cancer cases in the Iranian population. The present study found that the ASIR of all ocular cancers in Iran was 16.04 per 100,000 populations from 2004 to 2016. This study showed that there has been a slight increase in the ASIR of all ocular cancers in the 13-year period among both genders. However, the ASIR of total ocular cancers experienced a significant declining pattern between 2009 and 2016. Additionally, excepting retinoblastoma, a significant downward trend was observed in the age group over 50 years, males, ocular surface, and skin/canthus/adnexal cancers over the 13-year period. This analysis showed a minimal increasing trend in ASIR of all ocular cancers for the period of 2004–2016; which was in line with those reported from Zimbabwe^27^, the United States^22^, India^26^, Thailand^25^, Lebanon^24^, and the Asia–Pacific region^23^. However, the results of 18 registries in the United States from 2000 to 2011, showed that the incidence of oculars cancer decreased significantly in the age group over 40 years (APC = −1.7; p < 0.01)^30^.

In our study, the ASIR of total ocular cancers peaked in 2009. On the other hand, a significant downward trend was observed in the number of total ocular cancers, males, and those over 50 years of age from 2009 to 2016. The former upward trend prior to 2009 can be interpreted by improvements in registration protocols, updates in data quality control procedures, and adjustments in the reporting and/or categorization of malignancies^28^. The latter downward trend during the period from 2009 to 2016 may be due to advanced practice approaches and improved access to health care following the change in the INPCR system since 2009. We assume that in the recent years the relative reduction in the ASIR of ocular cancers reported by the INPCR may be due to the lack of pathological investigations on the cases that are treated according to the opinion of the ophthalmologists without histopathology results. For instance, choroidal melanoma is now managed by radioactive plaques^31,32^; and topical Gamma Interferon Alpha b2 is now used to treat OSSN^33^. In such cases, the globe will not be enucleated and the patient may die years later due to reasons not related to ocular cancers. Therefore, there may be no trace of such cancer incidences in classical pathology registries.

During the 13 years, the ASIR of all ocular cancers increased with age. In both genders, the highest incidence of ocular cancers was observed in those over 50 years of age, which may be attributed to increased environmental risk factors, improved screening, and increased life expectancy in Iran^34,35^. According to the results of the last census in Iran in 2016, 9.3% of the elderly population were 60 years and older^36^.The aging and growing population in Iran will exacerbate this status and may increase the incidence of cancer reported in the years to come^37,38^. Considering that, increase of age is a well-known risk factor that influences the incidence of a variety of ocular cancers^7–9,39^. The reason for this upward trend is most likely due to advances in medical diagnosis, increase of registered patients, and the improvement of the INPCR system in Iran^12^.

A study conducted with data from 1973 to 2009 in the United States showed that the overall age-adjusted incidence of cancers affecting the orbit, conjunctiva, and lacrimal glands was 3.39 (95%CI, 3.27–3.52) per million person-years and were most common in the over-50 age group at 9.51 (95%CI, 9.11–9.92) per million person-years^40^. In the present study, between the periods of 2004 and 2009, the overall ASIRs for ocular cancers increased in patients > 50 years of age. Changes in detection behavior and medical technologies may have played a role in the observed increase of ocular cancers in older patients^41–43^. However, the joinpoint regression analysis in our study showed that the overall ASIRs for ocular cancers decreased in patients under 50 years, which is likely related to the reduction in the cumulative effects of exposure to environmental risk factors. This can be explained by increased usage of sunglasses and brimmed hats that block UVB irradiation, use of sunshields in cars and home windows for UV protection, and implementation of safety instruments to minimize hazardous and toxic occupational exposures.

Since there is an important interaction between age, period, and cohort effect, we used an age–period–cohort analysis in our study. This analysis showed that the incidence of ocular cancers increased with age. The cancer rates at different ages were represented by the age effect. The results of the study showed that the RRs of ocular cancers increased rapidly with age. The longitudinal age curve of ocular cancers showed that the incidence rate of total ocular cancers was significantly increased at the age of 62.5 years. The local drift values did not show a fixed pattern; however, the overall net annual drift for the incidence of ocular cancers between 2004 and 2016 was 0.21% per year. Therefore, we should pay more attention to the prevention, control, and allocation of cancer treatment to the older age groups. These fluctuations that exist throughout the reporting of the overall trends of ocular cancers in Iran are mainly determined by demographic changes and aging of the population, as well as the socioeconomic status (SES)^44,45^, air pollutants^46,47^, rapid urbanization^48^, and improvement of preventive methods, treatment, and other diagnostic tests^49^.

Cohort effects represent changes between groups of individuals born in the same year^50^. These changes can be due to disparities that exist at the socioeconomic level and among environmental factors during the early life of individuals that impact on the risk of developing ocular cancers. The present study highlights the existence of a cohort effect in the incidence of ocular cancers in Iran from 2004 to 2016. It was found that the cohort effect on the incidence of ocular cancers was higher in the 2009 birth cohorts than in the previous cohorts. In the same age group, the incidence of ocular cancers increased from older cohorts to newer birth cohorts. The increase of RR in the birth cohort in 2009 reflects the introduction of an updated cancer registration system and protocols in the country. Previous studies have shown that an increase in cancer incidence may be attributed to a shift in coding or registration practices, changes in medical practice and improved diagnostic technologies^28,51^.

This study reported that the age-standardised incidence rate of all types of ocular cancers in Iran was 18.22 and 13.81 per 100,000 population for males and females, respectively. Males were more frequently affected than females by most of the ocular cancers. Similar to other studies^25,40,52,53^, our results showed that the overall incidence of ocular cancers peaked in males, while it continued to decrease in females. The increasing trend of ocular cancer incidence in Iranian males may be attributed to their prolonged outdoor exposure.

In addition, we assessed the ASIR trend of ocular cancers by topography and morphology types. In our study, carcinomas/adenocarcinomas were the most common types of ocular cancers that had a significant increasing trend between the years of 2004–2009; however, their trend was significantly decreasing from the years of 2009 to 2016. In a study in England^52^, eye lid squamous cell carcinoma had a significant upward trend over a 15-year period. In the current study, the ASIR trend of retinoblastoma showed a significant upward trend in both genders during the 13-year period. Our results were in line with two previously published papers, in which an increasing trend of retinoblastoma in Iranian children was report^54,55^. Similar results were observed in a study in Thailand, in which the incidence trend of retinoblastoma was upward by 2% annually between 1990 and 2009^25^. SY Li. et al., also found that the incidence of retinoblastoma in Taiwan increased slightly between 1998 and 2009 throughout the study period^56^. However, in a previous study in the United States (1975–2004)^19^, similar to European countries^57^, the ASIR of retinoblastoma remained stable over the study period.

The incidence of ocular cancers varied geographically during the 2004-to-2016 period, with the highest incidence observed in the West, and the lowest in the East and North of Iran. The incidence of ocular cancers was also highest in regions with low SES and low income^28,58,59^. Several studies have shown that individuals with lower SES have a higher incidence of body cancers^60,61^. Locating in latitudes of 25 to 40 degrees from the Equator exposes the general population of Iran to more UVB irradiation, as it has been shown that living within 10 degrees of latitude close to the Equator can double the risk of developing OSSN^10^. In a study by Moslehi et al^62^, the provincial comparison showed a general but inconsistent correlation between the UVB irradiation and melanoma incidence rate in Iran, where the highest incidence rates belonged to the Southern provinces of Iran, which are closer to the equator and have a high UVB exposure. Carcinogenic UVB irradiation along with an aging population are two major non-modifiable risk factors for developing various types of ocular malignancies^7–9,39,63^, which are of concern in Iran and demonstrate the importance of implementing community-based health policies to minimize the impact of environmental exposures and demographic characteristics on the development of ocular cancers in upcoming years. Health policies may include a comprehensive public education program about the menaces of sunlight (UVB) and the necessary and proper ways to protect against it., In addition, structural reforms in the cancer registry system are needed in order to optimize early diagnosis and treatment of patients.

Our study had some limitations. The cancer registry data did not include information on other risk factors associated with ocular cancer. Additionally, the present study only estimated the trend of all ocular cancers during the period of 2004-to-2016. However, the registry had a complete record of premium quality data, which was critical for establishing a comprehensive description of the trend in incidence. Future studies are recommended for continued evaluation of the disparities that exist in the characteristics of ocular cancers, focusing on socioeconomic and environmental risk factors that lead to the development of these cancers, along with the respective societal burdens, and assessing high-incidence groups in the hot spot regions.

## Conclusion

In summary, although ocular cancers are still considered rare in Iran, understanding its characteristics for future diagnosis is of great importance in ensuring good quality of life via proper healthcare policy management. Our findings suggest remarkable increasing incidence trends among older adults and retinoblastoma. The incidence rates of ocular cancers increase with age; the age effect may be a critical factor influencing the incidence of ocular cancers reported. The aging of the Iranian population and rapid urbanization could affect the incidence of ocular cancers reported due to the improved access to health care which assists with declining health issues and accurate registering of cases. This study was based on the acquired data from the INPCR, rather than a cohort study. Large cohort studies in different regions of the country are needed to determine relative cohort and period risks. Ocular cancer incidence trends and statuses inform ophthalmologists and health care decision-makers for future monitoring, resource allocation decisions, and planning. Future investigations should be continued in older age groups and high-risk regions.

## Materials and methods

### Data sources

We used ocular cancer incidence data acquired from INPCR. Cancer registry activities started in Iran in the 1950s, however, a formal registry started in 1986. During the early 2000s, with the collaboration of the Iranian Ministry of Health and Medical Education and local universities, population-based cancer registries were launched in Iran. The systematic ocular cancer registry was started since 2004 for each province^28^. Overall, 12,824 new ocular cancers were registered in INPCR between 2004 and 2016.

All ocular cancers from 2004 to 2016 and corresponding to the third edition of International Classification of Diseases (ICD-O-3) topography codes (44.1, 69.0–69.6, 69.8, and 72.3) were included. All cases were histopathologically reported according to ICD-O-3 as a basis for coding cancers. Only cases of verified histologies were enrolled in our study. All cancers growing directly from the globe, eyelids skin, conjunctiva, accessory lacrimal glands, and distant metastases to the orbit were included. Topographic location codes C69.6 (orbital) and C69.5 (lacrimal) were used to identify orbital cancers. Topographic codes C69.3 (choroid) and C69.4 (ciliary body) were used to filter out all uveal cancers, and C44.1 was used to indicate neoplasms of the eyelid including the skin/canthus/adnexa. Cancers were coded according to the morphology codes described in the ICD-O-3; codes of 801–856 for carcinomas/adenocarcinomas, 959–975 and 980–996 for lymphomas, 872–879 for melanomas, 880–909, 914, 925 and, 958 for sarcomas, 935–957, except 951 for neurologic cancers, 951 for retinoblastoma, and 800, 8806, 894, 906–909 and, 931 for mixed, rare and unspecified cancers^38^.

In INPCR, the patient’s national identification number, gender, age, province, diagnosis, grade, behavior, histology, and cancer location were recorded from diagnostic facilities and death certificates. Since the national identification number is unique for each resident in Iran, duplicates could be checked and eliminated. A detailed description of the data sources, data processing, and compilation can be found elsewhere^28^. Informed consent was not required as the reported data was anonymous, and patients’ personal data was not used in any stage of the analysis Ethical approval was obtained (IR.SBMU.ORC.REC.1401.010) from the Ethics Committee of the Ophthalmic Research Center at the Research Institute for Ophthalmology and Vision Science, Shahid Beheshti University of Medical Sciences, Tehran, Iran, and all methods were performed under the relevant guidelines and regulations.

### Statistical analyses

In the present study, we estimated age-specific (5-year age groups) and crude incidence rates per 100,000 persons for each year and by gender, age group at diagnosis (< 50 and  ≥ 50 years), topography, and morphology types. The number of ocular cancer cases in 17 age groups by gender, types of topography and morphology formed the basis for the calculation of age standardisation. Age standardisation was performed by the direct method using the latest World Bank databank and standardised the population for each year^64^. The age-standardised incidence rate (ASIR) for an age group composed of ages x through y is determined using the National Cancer Institute (NCI) formula^14^.Given that 41% of all patients in the INPCR data were over 50 years of age, we chose “50” years for categorising the age group at diagnosis. The incidence rates and relative risks were calculated for each of the age, topography and morphology groups.

Temporal trends in the ASIR were examined using joinpoint regression, with a maximum of two joinpoints and with changes in trend expressed as an APC^65^ and AAPC^66^, assuming a constant rate of change in the logarithm of the annual ASIR in each segment. For 13 data points, the joinpoint software recommended a maximum of one joinpoint. We used the Bayesian information criterion method and significant change in the linear slope of the trend for the best-fitting points^67^. This was performed starting with a minimum of zero i.e., a straight line. APC was analysed by gender, age group at diagnosis, topography, and morphology types (2004–2016). The significance of APC was tested using an asymptotic t-test and considered significant at 5%.

In the present study, the age-period-cohort model was used to analyse the effects of age, period (year of diagnosis), and cohort (year of birth) on change trends in ocular cancer incidence. This model was fitted to the crude incidence rates using the age-period-cohort analysis web tool^4^. Individuals’ age was divided into 17 five-year intervals (0–4, 5–9, …, 75–79, ≥ 80 years). The year of diagnosis was divided into 3 categories (2004–2008, 2009–2013, and 2014–2016). The cohort was indexed by midyear of birth (1924, 1929, …, 2009, 2014). Reference age, period, and cohort were defined as the median of each range. Five age-period cohort parameters and functions were calculated and presented in the study, including net drift, local drift, longitudinal age curve, period RR, and cohort RR^68^.

Provincial ASIR was also calculated and reported based on reported data during the thirteen years (2004–2016). Provincial ASIR was presented in three groups based on percentiles (33.3 and 66.7). The Getis-Ord Gi and Moran's I index were used to test the ASIR variations and spatial autocorrelation between provinces, considering that the results of these tests were significant, the spatial Getis–Ord statistic (Gi*) was used to identify the geographical hot spots and cold spots of ASIR throughout the country^69,70^.

Data preparation was performed in Stata software; in the first part of the study which aimed to assess the incidence rate and relative risk, 0.7% of the “age at diagnosis” variable was missing. Before the trend analysis, the full-information maximum likelihood (FIML) estimation was used for handling missing data of this variable^71^.

Statistical analyses were performed using Microsoft Excel 2016 (Microsoft Corporation, Redmond, WA, USA), Stata version 17.0 (StataCorp, College Station, TX, USA), ‘Joinpoint’ software (Joinpoint Regression Program, version 4.8.0.1, NCI) provided by the US National Cancer Institute, the age-period-cohort web tool provided by the National Cancer Institute, and ArcGIS software, version 10.3 (ESRI, Redlands, CA, USA). A p-value < 0.05 was considered statistically significant.

### Ethics approval

Ethical approval was obtained (IR.SBMU.ORC.REC.1401.010) from the Ethics Committee of the Ophthalmic Research Center affiliated to Shahid Beheshti University of Medical Sciences, Tehran, Iran.

## Supplementary Information

Supplementary Information.

## Data Availability

The datasets generated and analyzed during the current study are available from the corresponding author upon reasonable request.
