# Publisher Correction: A joinpoint and age–period–cohort analysis of ocular cancer secular trends in Iran from 2004 to 2016

**DOI:** 10.1038/s41598-023-29520-0

**Published:** 2023-02-14

**Authors:** Mohammad Abolhosseini, Zahra Khorrami, Sare Safi, Mohammad Esmaeil Akbari, Seyed Mohamadmehdi Moshtaghion, Seyed Farzad Mohammadi, Mozhgan Rezaei Kanavi, Saeed Karimi

**Affiliations:** 1 Ophthalmic Epidemiology Research Center, Research Institute for Ophthalmology and Vision Science, Shahid Beheshti University of Medical Sciences, No. 23, Paidarfard St., Pasdaran Ave., Tehran, Iran; 2 Ocular Tissue Engineering Research Center, Research Institute for Ophthalmology and Vision Science, Shahid Beheshti University of Medical Sciences, No 23, Paydar fard st, Pasdaran ave, Tehran, Iran; 3 Cancer Research Center, Shahid Beheshti University of Medical Sciences, Tehran, Iran; 4 Ophthalmic Research Center, Research Institute for Ophthalmology and Vision Science, Shahid Beheshti University of Medical Sciences, No 23, Paydar fard st, Pasdaran ave, Tehran, Iran; 5 Translational Ophthalmology Research Center, Farabi Eye Hospital, Tehran University of Medical Sciences, Tehran, Iran

Correction to: *Scientific Reports* 10.1038/s41598-022-26349-x, published online 19 January 2023

The original version of this Article contained an error in the order of the author names, which was incorrectly given as Mohammad Abolhosseini, Zahra Khorrami, Sare Safi, Mohammad Esmaeil Akbari, Seyed Mohamadmehdi Moshtaghion, Seyed Farzad Mohammadi, Saeed Karimi & Mozhgan Rezaei Kanavi.

The original Article has been corrected.

