# Supplementary material for: A joinpoint and age–period–cohort analysis of ocular cancer secular trends in Iran from 2004 to 2016
Source: Sci Rep. 2023 Jan 19;13:1074. doi: 10.1038/s41598-022-26349-x (PMC9852578; doi:10.1038/s41598-022-26349-x)
Supplement: Supplementary file 1 — Supplementary Information. [file 41598_2022_26349_MOESM1_ESM.docx]

**Supplementary files**


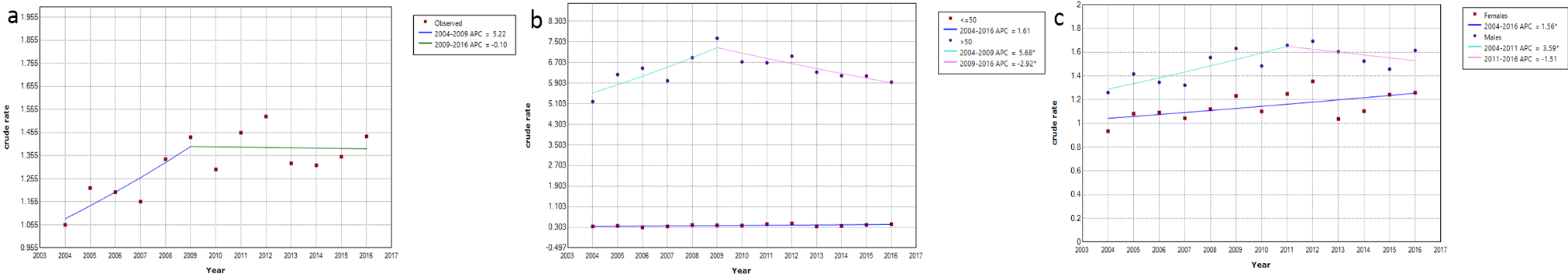


Figure S1: The trend in incidence crude rate (world population, per 100,000 population) incidence rates for (a) Ocular cancers overall, (b) by age group, and (c) by gender in Iran between 2004 and 2016 (trend modeled with joinpoint regression).

Table S1. Annual crude incidence rates of ocular cancers in Iran between 2004 and 2016.

|  | **Trend 1** | | **Trend 2** | |  |
| --- | --- | --- | --- | --- | --- |
|  | **Period** | **APC (95% CI)** | **Period** | **APC (95% CI)** | **2004-2016**  **AAPC (CI95%)** |
| **Overall**** | 2004-2009 | 5.2(0.2, 10.9) * | 2009-2016 | -0.1(-2.8, 2.7) | 2.1(-0.3, 4.5) |
| **Age group** |  |  |  |  |  |
| ≤50 year** | 2004-2009 | 1.6(-0.1, 3.4) |  |  | 1.6(-0.1, 3.4) |
| >50 year | 2004-2016 | 5.7(3.2, 8.2) * | 2009-2016 | -2.9(-4.9, -0.9) * | 0.6(-0.8, 1.9) |
| **Gender** |  |  |  |  |  |
| Males** | 2004-2016 | 1.6(0.1, 0.3) * |  |  | 1.6(0.1, 0.3) * |
| Females | 2004-2011 | 3.6(0.7, 6.6) * | 2011-2016 | -1.5(-5.6, 2.8) | 1.4(-0.6, 3.6) |
| **Topography** |  |  |  |  |  |
| Orbit | 2004-2012 | 9.8(0.7, 19.7) * | 2012-2016 | -3.6(-20.6, 17) | 5.2(-2.3, 13.2) |
| Lacrimal Gland and Duct | 2004-2007 | -13.5(-51.2, 53.3) | 2007-2016 | 2.6(-6.2, 12.3) | -1.7(-14.1, 12.5) |
| Intraocular** | 2004-2016 | -2.4(-5.1, 0.4) |  |  | -2.4(-5.1, 0.4) |
| Ocular Surface | 2004-2011 | 1.1(-3.7, 6.3) | 2011-2016 | -12.1(-20, -3.4) * | -4.6(-8.5, -0.6) * |
| Skin / Canthus / Adnexa | 2004-2009 | 4.6(0.3, 9.1) * | 2009-2016 | 0.7(-1.5, 2.9) | 2.3(0.4, 4.2) * |
| Unspecified** | 2004-2016 | 3.3(-0.7, 7.5) |  |  | 3.3(-0.7, 7.5) |
| **Morphology** |  |  |  |  |  |
| Carcinoma / Adenocarcinoma | 2004-2011 | 3.5(1, 6.1) * | 2011-2016 | -2.5(-4.7, -0.3) * | 1(-0.5, 2.4) |
| Lymphoma | 2004-2012 | 14.2(2.3, 27.4) * | 2012-2016 | -9.1(-29.5, 17.2) | 5.8(-3.8, 16.4) |
| Melanoma** | 2004-2016 | -0.9(-3.7, 2) |  |  | -0.9(-3.7, 2) |
| Sarcoma** | 2004-2016 | 6.6(2, 11.4) * |  |  | 6.6(2, 11.4) * |
| Neurologic** | 2004-2016 | 5.3(-5.3, 17) |  |  | 5.3(-5.3, 17) |
| Retinoblastoma** | 2004-2016 | -1.3(-4.3, 1.8) |  |  | -1.3(-4.3, 1.8) |
| Others (Mixed, Rare and Unspecified) ** | 2004-2016 | 40.4(29.8, 52) * |  |  | 40.4(29.8, 52) * |

Note: APC: annual percentage change; AAPC: average annual percent change; CI: confidence interval;

* The annual percent changes (APC) were significantly different from 0 for a specific trend (P-value<0.05).

** The linear model without a joinpoint best describes the trend.
